# Neural Induction in *Xenopus*: Requirement for Ectodermal and Endomesodermal Signals via Chordin, Noggin, β-Catenin, and Cerberus

**DOI:** 10.1371/journal.pbio.0020092

**Published:** 2004-05-11

**Authors:** Hiroki Kuroda, Oliver Wessely, E. M. De Robertis

**Affiliations:** **1**Department of Biological Chemistry, Howard Hughes Medical InstituteUniversity of California, Los Angeles, CaliforniaUnited States of America

## Abstract

The origin of the signals that induce the differentiation of the central nervous system (CNS) is a long-standing question in vertebrate embryology. Here we show that *Xenopus* neural induction starts earlier than previously thought, at the blastula stage, and requires the combined activity of two distinct signaling centers. One is the well-known Nieuwkoop center, located in dorsal-vegetal cells, which expresses Nodal-related endomesodermal inducers. The other is a blastula *Chordin-* and *Noggin-*expressing (BCNE) center located in dorsal animal cells that contains both prospective neuroectoderm and Spemann organizer precursor cells. Both centers are downstream of the early β-Catenin signal. Molecular analyses demonstrated that the BCNE center was distinct from the Nieuwkoop center, and that the Nieuwkoop center expressed the secreted protein Cerberus (Cer). We found that explanted blastula dorsal animal cap cells that have not yet contacted a mesodermal substratum can, when cultured in saline solution, express definitive neural markers and differentiate histologically into CNS tissue. Transplantation experiments showed that the BCNE region was required for brain formation, even though it lacked CNS-inducing activity when transplanted ventrally. Cell-lineage studies demonstrated that BCNE cells give rise to a large part of the brain and retina and, in more posterior regions of the embryo, to floor plate and notochord. Loss-of-function experiments with antisense morpholino oligos (MO) showed that the CNS that forms in mesoderm-less *Xenopus* embryos (generated by injection with *Cerberus-Short* [*CerS*] mRNA) required Chordin (Chd), Noggin (Nog), and their upstream regulator β-Catenin. When mesoderm involution was prevented in dorsal marginal-zone explants, the anterior neural tissue formed in ectoderm was derived from BCNE cells and had a complete requirement for Chd. By injecting *Chd* morpholino oligos (*Chd-*MO) into prospective neuroectoderm and Cerberus morpholino oligos (*Cer-*MO) into prospective endomesoderm at the 8-cell stage, we showed that both layers cooperate in CNS formation. The results suggest a model for neural induction in *Xenopus* in which an early blastula β-Catenin signal predisposes the prospective neuroectoderm to neural induction by endomesodermal signals emanating from Spemann's organizer.

## Abstract 2

New insights into the early events of neural induction which render certain cells competent to respond to signals emanating from Spemann's organizer

## Introduction

Vertebrate development results from a series of cell–cell interactions in which groups of cells induce their neighbors to acquire new cell differentiation fates. This process, known as embryonic induction, was first reported for the induction of the lens in surface ectoderm by the optic vesicles originating from the brain (Spemann 1901; Lewis 1904). Subsequent work showed that the surface ectoderm itself also plays an important role (reviewed by Grainger 1992). From the analysis of lens induction, Spemann (1938) proposed that a double assurance mechanism (*doppelte Sicherung*) could provide a way of explaining the robustness of vertebrate development via reciprocal interactions between two layers of cells. Lens induction is an example of a secondary embryonic induction. Most experimental embryologists concentrated their research on the induction of the neural plate, which is considered the primary embryonic induction (Spemann 1938; Saxén and Toivonen 1962; Harland 2000; Gilbert 2001; Stern 2002). In the classical organizer transplantation experiment, Spemann and Mangold (1924) demonstrated that dorsal lip mesoderm is sufficient to induce the differentiation of a complete central nervous system (CNS) in responding ectoderm. Spemann devoted an entire chapter of his book to the discussion of whether a double assurance mechanism existed in the case of neural plate induction (Chapter 8 in Spemann 1938) and concluded that the evidence supported a role for the underlying mesoderm, but not for the prospective neuroectoderm.

**Figure 6:**
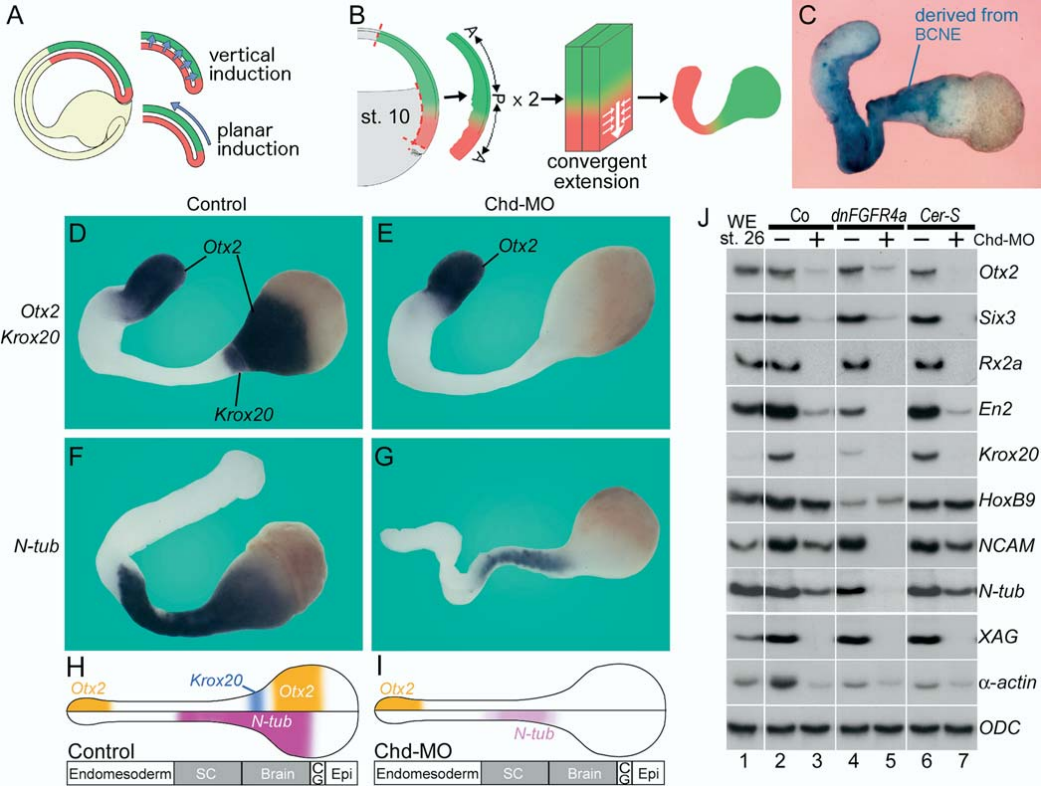
Anterior Neural Induction in Keller Explants Requires Chd (A) Proposed vertical and planar signals in neural induction (following Ruiz i Altaba 1993). (B) Diagram of Keller explant preparation and subsequent elongation of the endomesoderm by convergent extension (Keller 1991). (C) The neural and mesodermal regions of Keller explants contain descendants of BCNE cells (in blue) marked by blastomere injection at the 64-cell stage. (D) Expression of *Otx2* and *Krox20* in Keller explants (*n* = 7). (E) Injection of 17 ng *Chd-*MO completely blocked *Otx2* and *Krox20* expression in neural regions, while expression of *Otx2* in anterior endoderm was not affected (*n* = 10). (F) The differentiated neuron marker *N-tubulin* is expressed in Keller explants (*n* = 8). (G) Partial inhibition of *N-tubulin* by injection of *Chd-*MO (*n* = 7). (H and I) Summary of the effects of *Chd-*MO in Keller explants. Abbreviations: SC, spinal cord; CG, cement gland; Epi, epidermis. (J) RT-PCR analyses of the effect of *Chd-*MO in Keller explants; samples injected with (plus) or without (minus) *Chd-*MO are indicated. Lane 1, whole embryos; lanes 2–7, Keller sandwiches. Note that expression of the neural markers *NCAM* and *N-tubulin* in Keller sandwiches was abolished by co-injection with 200 pg of dnFGF receptor 4a (*dnFGF4a*) mRNA and 17 ng of *Chd-*MO (lane 5). Injection with 600 pg of *CerS* mRNA, which eliminates mesoderm but not BCNE formation, does not affect neural induction in this assay (lane 6).

A role for the gastrula ectoderm in neural plate formation had been proposed on the basis of experiments in which the mesoderm or the ectoderm had been damaged (Goerttler 1925) and received some subsequent support (Lehmann 1928). However, further consideration of the possible role of ectoderm in neural plate formation was hampered by a highly influential exogastrulation experiment performed in axolotl embryos (Holtfreter 1933), in which endomesoderm involution was prevented and the entire ectoderm differentiated into epidermis. Since there was no trace of CNS tissue in these embryos, this experiment was interpreted as a demonstration that the underlying endomesoderm had the essential role in neural plate induction and that the prospective neuroectoderm had none (Holtfreter 1933; Spemann 1938). The debate concerning whether the ectoderm itself has a role in neural plate formation has continued to this day. In dorsal marginal zone explants (Keller and Danilchik 1988; Keller 1991), CNS differentiation can take place in the absence of underlying mesoderm. It has been proposed that in these Keller explants neural tissue induction results from a “planar” signal that diffuses in the plane of the ectoderm from the mesodermal organizer at gastrula (Ruiz i Altaba 1992, 1993; Doniach et al. 1992; Poznanski and Keller 1997) (see Figure 6A). However, the existence of planar neural induction signals has been disputed, with neural induction in Keller explants proposed to result from “vertical” signals resulting from a brief contact between ectoderm and mesoderm at early gastrula (Nieuwkoop and Koster 1995). Therefore, a central question remains unanswered despite many decades of research in amphibian neural induction: What is the differentiation potential of the presumptive neural plate material in the absence of a mesodermal substratum? This is the problem addressed here.

Two recent technical advances led us to reinvestigate neural induction in *Xenopus*. First, it is now possible to completely inhibit mesoderm formation by microinjecting *Cerberus-short* (*CerS*) mRNA, a secreted antagonist specific for Nodal-related mesoderm inducers (Agius et al. 2000). Interestingly, *Xenopus* embryos lacking mesoderm still developed a CNS, including a cyclopic eye (Wessely et al. 2001). This was surprising, because such mesoderm-less embryos did not express multiple Spemann organizer markers such as *Chordin* (*Chd*), *Noggin* (*Nog*), and *Goosecoid* in dorsal endomesoderm at the gastrula stage. Second, a technical revolution has taken place with the availability of antisense morpholino oligos (MO) that permit loss-of-function studies in *Xenopus* (Heasman et al. 2000). It is now possible to combine the tools of amphibian experimental embryology with investigations on the role of individual genes, such as the secreted bone morphogenetic protein (BMP) antagonist Chd (Oelgeschläger et al. 2003) or its upstream regulator β-Catenin (Heasman et al. 2000), in experimentally manipulated embryos.

In whole embryos injected with *Chd-*MO, a CNS, although of reduced size, still develops. However, Spemann organizers depleted for Chd lose all neural-inducing activity when grafted to the ventral side of a host embryo (Oelgeschläger et al. 2003). Surprisingly, when similar Chd-depleted grafts are placed on the dorsal side, ectodermal cells lose the ability to contribute to neural plate (Oelgeschläger et al. 2003). This suggested that a cell-autonomous requirement of Chd for neural plate formation might exist in the ectoderm itself. At the blastula stage, the BMP antagonists *Chd* and *Nog* are expressed in the dorsal animal cap and marginal zone, in a region we had originally designated as the “preorganizer center” (Wessely et al. 2001). This group of cells constitutes a blastula *Chordin*- and *Noggin*-expressing (BCNE) region that contains both prospective neuroectoderm cells and Spemann organizer precursors. The BCNE region also expresses *Xenopus* Nodal-related 3*(Xnr3)*, a secreted factor with neural-inducing properties that is expressed at high levels in early *Xenopus* embryos (Haramoto et al. 2004; Wessely et al. 2004). The early phase of expression of *Chd* and *Nog* in BCNE cells is regulated by the dorsal accumulation of β-Catenin, whereas later expression of the same genes in Spemann organizer endomesoderm requires in addition Nodal-related signals that can be blocked by *CerS* (Wessely et al. 2001).

In this study we analyze the mechanism of neural induction in *Xenopus* by means of embryological cut-and-paste and molecular loss-of-function experiments. We find that the BCNE center contains much of the presumptive anterior CNS. Loss-of-function studies show that gene products expressed at blastula—such as Chd, Nog, and β-Catenin—are required for neural induction in the absence of underlying endomesoderm. Cell-lineage studies show that the BCNE center itself gives rise to brain, notochord, and floor plate. Transplantation experiments show that the BCNE center is required for brain formation in *Xenopus* embryos. Microinjection experiments at the 8-cell stage, in which *Chd-*MO was injected into dorsal-animal and *Cer-*MO into dorsal-vegetal blastomeres, confirmed that secreted signals from both prospective neuroectoderm and underlying endomesoderm are required for anterior CNS development. The results support a double assurance mechanism for brain formation of the type proposed by Spemann (1938) for lens induction.

## Results

### The BCNE Center Is Distinct from the Nieuwkoop Center

**Figure 1:**
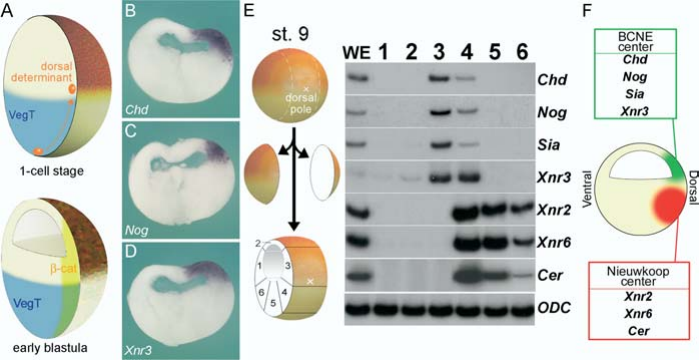
Two Signaling Centers Coexist in the *Xenopus* Blastula (A) Diagram of early events between 1-cell stage and early blastula. (B–D) Expression of *Chd, Nog,* and *Xnr3* transcripts just after midblastula transition (7 h postfertilization). Embryos were hybridized as whole mounts, stored in methanol for 1 mo at room temperature to improve contrast, and sectioned with a razor blade. (E) RT-PCR analysis of gene markers at midblastula, early stage 9. Six samples were prepared by dissections of blastula regions as shown in the diagram. (F) Summary of gene expression at blastula. The BCNE center expresses *Chd, Nog, Siamois,* and *Xnr3,* while the Nieuwkoop center expresses *Xnr2, Xnr6,* and *Cer.*

**Figure 2:**
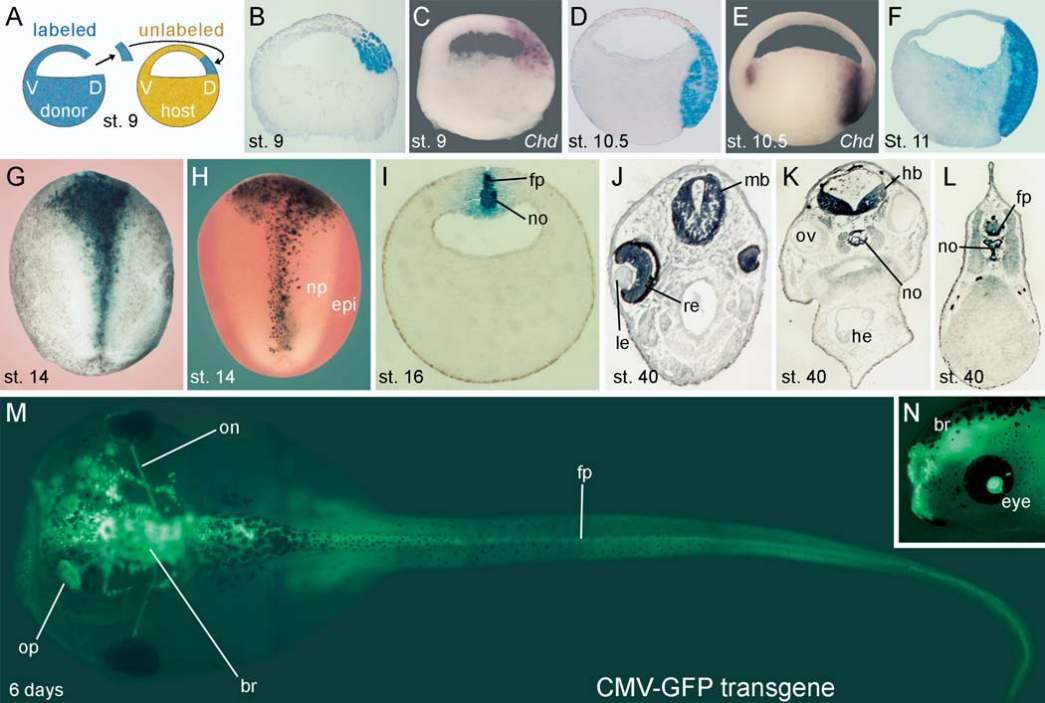
The BCNE Center Contributes to Forebrain and Midline Structures (A) Method used for lineage tracing of the BCNE center with biotin-dextran amine (BDA) labeled grafts. (B) Sagittal section of a recently grafted BCNE at stage 9. (C) *Chd* mRNA expression at stage 9. (D) BCNE descendants at stage 10.5. (E) *Chd* mRNA expression at stage 10.5. (F) BCNE center descendants at stage 11. (G) Dorsal view of BCNE descendants at neural plate stage 14. (H) Double staining of transplanted BCNE region with nuclear *lacZ* mRNA and epidermal ectoderm of the host with epidermal cytokeratin (epi) probe in light red at stage 14. (I) Transverse section at the level of the trunk at stage 16. Abbreviations: fp, floor plate; no, notochord. (J–L) Transverse sections at stage 40. Abbreviations: fp, floor plate; hb, hindbrain; he, heart; le, lens; mb, midbrain; no, notochord; ov, otic vesicle; re, retina. (M) Dorsal view of 6-d embryo transplanted with a BCNE graft from CMV-GFP transgenic embryos. Abbreviations: br, brain; fp, floor plate; on, optic nerve; op, olfactory placode. (N) Side view at 4 d showing labeled retina and brain. Abbreviation: br, brain.

The initial asymmetry in *Xenopus* development is caused by a cortical rotation triggered by sperm entry, thought to redistribute “dorsal determinants” that in turn stabilize β-Catenin protein on the dorsal side of the embryo (Figure 1A) (reviewed in Gerhart et al. 1991; Harland and Gerhart 1997; De Robertis et al. 2000). At the blastula stage, the Nieuwkoop center located in dorsal-vegetal cells secretes mesoderm-inducing signals such as *Xnr1, Xnr2, Xnr4, Xnr5,* and *Xnr6* that induce formation of the gastrula Spemann organizer in overlying mesoderm cells (Agius et al. 2000; Takahashi et al. 2000). The Nieuwkoop center has also been called the “blastula organizer” in the early literature (Gerhart et al. 1991). The BCNE region develops in the dorsal animal and marginal region. In situ hybridization analyses at the blastula stage (7 h after fertilization) showed that the neural-inducing secreted factors *Chd, Nog,* and *Xnr3* are expressed in the animal cap, in a region that includes about 45^o^ of arc above the floor of the blastocoel, as well as in the dorsal marginal zone (Figure 1B–1D). At the gastrula stage, the same genes are expressed in more vegetal regions, in the Spemann organizer located in the dorsal endomesoderm of the marginal zone (e.g., Figure 2E).

The question arises as to whether two distinct signaling centers coexist in the *Xenopus* blastula. To address this, early blastulae with strong dorsoventral polarity (Klein 1987) were dissected into six fragments, as shown in Figure 1E. The results showed that, although some overlap existed, the region expressing *Chd* and *Nog* included the animal cap, whereas the Nieuwkoop center region that expresses *Xnr2* and *Xnr6* had a more vegetal location (see Figure 1E). *Xnr3* expression was observed in fragments 3 and 4, indicating a higher degree of overlap (see Figure 1E). In addition, the results showed that the homeobox gene *Siamois* was expressed in the BCNE region, even though its expression has been reported to be more vegetal at later stages of development (Lemaire et al. 1995). We also found that *Cerberus (Cer),* a gene expressed in anterior endoderm at gastrula (Bouwmeester et al. 1996), was a component of the Nieuwkoop center. We conclude that two distinct signaling centers are present at blastula (see Figure 1F). The more animal BCNE center expresses *Chd, Nog, Xnr3,* and *Siamois,* whereas the Nieuwkoop center expresses *Xnr2, Xnr6,* and *Cer.*

### Cell Lineage of the BCNE Region

To map the fate of the blastula *Chd-* and *Nog-*expressing cells during normal development, we transplanted lineage-labeled BCNE regions isotopically into host blastulae at early stage 9 (Figure 2A). These grafts containing the lineage tracer biotin-dextran amine (BDA) marked the *Chd-*expressing region at blastula (compare Figure 2B and 2C). A few hours later, at early gastrula (stage 10.5), dorsal animal cap descendants were found both in organizer endomesoderm and in prospective neuroectoderm (Figure 2D). We note that by early gastrula stage *Chd* mRNA was expressed in organizer endomesoderm, but was no longer detectable in prospective neuroectoderm (Figure 2E). At midgastrula, stage 11, the transplanted tissue elongated in organizer endomesoderm and prospective neuroectoderm, with both layers remaining in close apposition (Figure 2F). At neural plate stages, stage 14, BCNE center descendants were found in a wide region in the anterior neural plate and, more posteriorly, in a narrow stripe in the midline (Figure 2G). Double staining using nuclear *lacZ* mRNA as lineage tracer in combination with an epidermal cytokeratin marker confirmed that BCNE cells give rise to anterior neural plate (Figure 2H). The midline staining in the trunk region corresponded to floor plate and notochord in histological sections (Figure 2I). At tadpole stage (stage 40), BCNE descendants contributed to a large part of the brain and retina (but not lens and otic vesicles) and to dorsal midline structures of the trunk-tail region (Figure 2J–2L). This lineage could be traced up to feeding tadpole stages (Figure 2M and 2N) using dorsal animal cap grafts from cytomegalovirus–green fluorescent protein transgenic embryos (Marsh-Armstrong et al. 1999). These results indicate that blastula *Chd-*expressing cells give rise to much of the brain and to the floor plate and notochord in the trunk region of the *Xenopus* embryo.

### The Dorsal Animal Cap Is Specified to Form CNS

**Figure 3:**
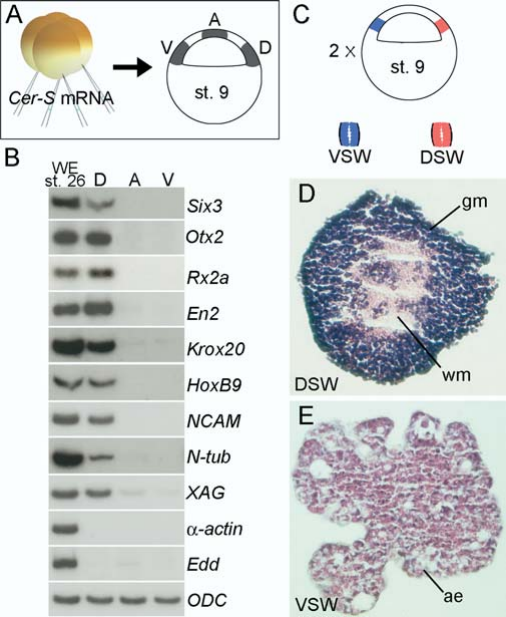
The Blastula Dorsal Animal Cap Is Specified to Form CNS (A) Experimental diagram showing embryos injected with *CerS* mRNA from which three regions of the animal cap were dissected at blastula, cultured until stage 26, and processed for RT-PCR. The size of the explants was 0.3 mm by 0.3 mm in these samples. Abbreviations: A, animal pole; D, dorsal region; V, ventral animal cap. (B) RT-PCR analysis of animal cap fragments; note that anterior brain markers were expressed in the dorsal fragments in the absence of mesoderm (*α-actin*) and endoderm (*endodermin, Edd*) differentiation. Abbreviations: A, animal pole; D, dorsal region; V, ventral animal cap. (C) Experimental diagram of the small animal cap sandwich experiments; these embryos were not injected with *CerS.* In this case, the size of the explants was 0.15 mm by 0.15 mm leaving a 0.15-mm gap from the floor of the blastocoel to avoid contamination from mesoderm-forming cells. Fragments from two explants were sandwiched together (explants are too small to heal by curling up) and cultured in 1× Steinberg's solution until stage 40. Abbreviations: VSW, ventral sandwich; DSW, dorsal sandwich. (D) Histological section of dorsal animal cap explant (dorsal sandwich). These sandwiches differentiated into histotypic forebrain tissue including white and gray matter (4/17). Abbrevations: DSW, dorsal sandwich; gm, gray matter; wm, white matter. (E) Histological section of a ventral animal cap sandwich. All sandwiches differentiated into atypical epidermis (*n* = 20). Abbreviations: ae, atypical epidermis; VSW, ventral sandwich.

In embryology, the test of whether cells are specified to form a particular tissue is to culture them in isolation from the rest of the embryo. Dorsal animal cap explants from embryos injected with *CerS* mRNA expressed multiple neural molecular markers at stage 26, whereas animal or ventral explants did not (Figure 3A and 3B). *CerS* was required to inhibit mesoderm formation; when identical explants were prepared without *CerS* mRNA injection, mesoderm contamination from the marginal zone was detected (data not shown). Neural differentiation could also be obtained in the absence of *CerS* mRNA when additional care was taken to avoid mesodermal contamination. As shown in Figure 3C, small explants from the top half of the BCNE region were excised, sandwiched together, and cultured in saline solution for 3 d. The sandwich procedure allows such small explants to survive in culture for long periods of time. Dorsal BCNE explants differentiated into histotypic CNS, including gray and white matter (Figure 3D), whereas similar explants from ventral ectoderm differentiated into epidermis (Figure 3E). These results demonstrate that dorsal animal cap cells are already specified to form CNS at the blastula stage.

### BCNE Tissue Is Required for Brain Formation

**Figure 4:**
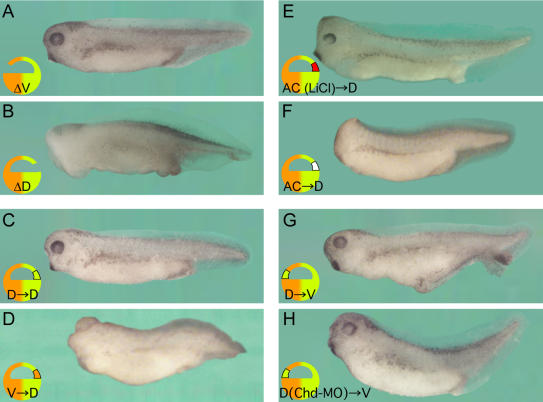
The Dorsal Animal Cap Is Required for Brain Formation (A) Ventral animal cap deletion (ΔV) produces a normal embryo. (B–F) Dorsal animal cap deletion (ΔD) results in loss of anterior brain structure. The headless phenotype of dorsal animal cap deletions was rescued by dorsal animal cap grafts (C) and animal pole grafts obtained from LiCl-treated embryos (E), but not by ventral animal cap transplants (D) or animal pole transplants (F). The average dorso-anterior indices (DAI) were 4.89 ([A] *n* = 28), 3.52 ([B] *n* = 25), 4.90 ([C] *n* = 10), 3.63 ([D] *n* = 19), 4.90 ([E] *n* = 12), and 3.50 ([F] *n* = 10). (G) Transplantation of the dorsal animal cap into the ventral animal cap region of a host embryo induced weak secondary axes (65.4%, *n* = 26). The embryo shown here was one of the strongest axes obtained. (H) Activity of BCNE transplanted ventrally was blocked by *Chd-*MO (*n* = 15).

To test whether the BCNE center is required for brain formation, we first deleted ventral or dorsal regions of the animal cap. Deletion of the dorsal region, but not of the ventral animal cap, resulted in headless embryos (Figure 4A and 4B). Since *Xenopus* is one of the best-studied vertebrate embryos, it was surprising that this requirement of a region of the blastula for CNS formation had not been reported previously. To investigate this further, we replaced the deleted fragment with various ectodermal grafts. The brain defects could be rescued by transplantation of dorsal, but not ventral, animal cap grafts (Figure 4C and 4D). Ectoderm from the animal pole was unable to rescue the ablated dorsal animal cap (Figure 4F). However, similar animal poles from lithium chloride (LiCl)–treated embryos, in which β-Catenin is stabilized and transcription of BCNE genes activated, were able to rescue head formation (Figure 4E).

Despite this requirement for brain development, blastula dorsal animal caps grafted into the ventral side of a host blastula were only able to form weak secondary axes (Figure 4G). *Chd-*MO, which blocks the activity of Spemann grafts (Oelgeschläger et al. 2003), also inactivated BCNE grafts (Figure 4H). However, an important difference with the mature Spemann organizer was that BCNE cell transplants self-differentiated into spinal cord and muscle in these weak axes and were unable to induce CNS in neighboring cells as the Spemann organizer does (data not shown). We conclude that the dorsal animal cap BCNE center is required for brain formation. However, when transplanted into ectopic sites, BCNE tissue has only weak effects and does not induce neural tissue.

### Anterior CNS Formation in the Absence of Mesoderm Requires Chd and Nog

**Figure 5:**
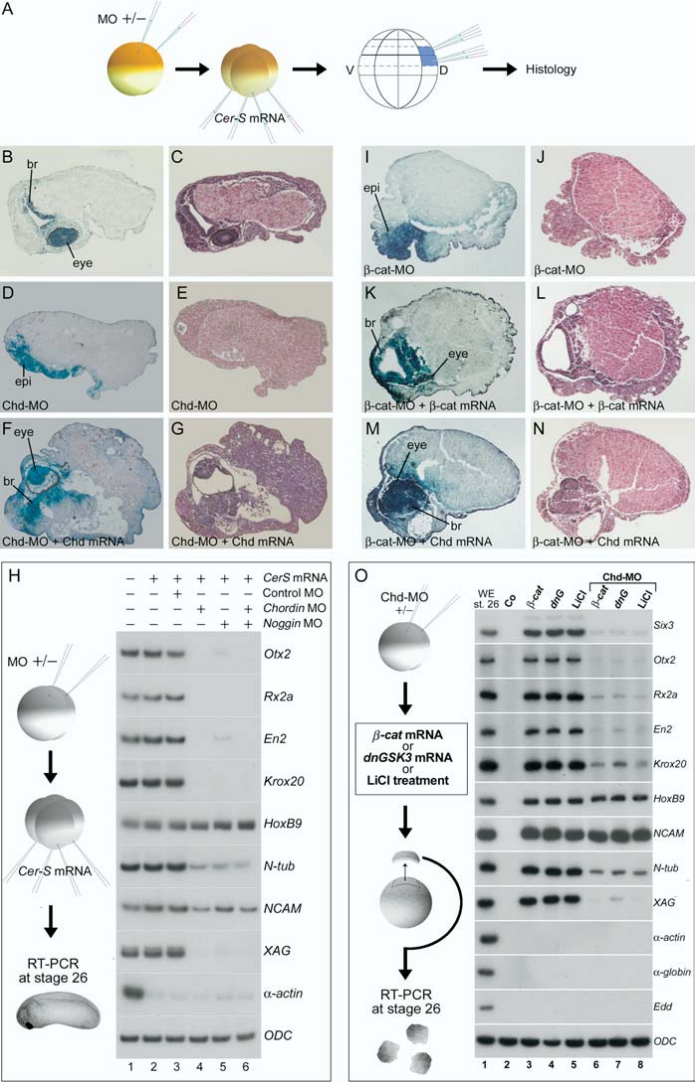
The CNS of Mesodermless Embryos Derives from BCNE Cells and Requires Chd, Nog, and β-Catenin (A) Experimental design. Embryos in which mesoderm induction was inhibited (by injection of 600 pg of *CerS* mRNA into the vegetal pole) were sectioned at stage 38 and stained with hematoxylin-eosin or for microinjected BDA lineage tracer marking the BCNE region. (B and C) Embryos injected with *CerS* mRNA alone (*n* = 40). Abbreviation: br, brain. (D and E) Embryos injected with 17 ng of *Chd-*MO in addition to *CerS* (*n* = 21). Abbreviation: epi, epidermis. (F and G) Coinjection of 17 ng of *Chd-*MO and *CerS,* followed by 100 pg of *Chd* mRNA together with the lineage tracer (*n* = 19). Abbreviation: br, brain. (H) Expression of anterior CNS markers in mesodermless embryos requires Chd and Nog. RT-PCR analysis of *CerS* mRNA–injected embryos at tailbud stage 26. Markers of anterior brain (*Otx2*), eye (*Rx2a*), midhindbrain boundary (*En2*), hindbrain (*Krox20*), and cement gland (*XAG*) were inhibited by injection of *Chd-*MO, *Nog-*MO, or both. A pan-neural marker (NCAM) and a neuronal marker (N-tubulin) were partially inhibited, and the posterior neural marker *HoxB9* was not affected. *α-actin* serves as a mesoderm marker to show that *CerS* blocked mesoderm in these embryos and *ODC* as mRNA loading control. The effects of the *Nog-*MO described here can be rescued by full-length *Nog* mRNA lacking the 5′ leader sequence targeted by the antisense morpholino (data not shown). (I and J) *β-cat-*MO (13.6 ng) together with *CerS* mRNA (*n* = 15). Abbreviation: epi, epidermis. (K and L) Rescue of *β-cat-*MO by 800 pg of *β-catenin* mRNA. Abbreviation: br, brain. (M and N) Rescue of the *β-cat-*MO phenotype by 100 pg of *Chd* mRNA (*n* = 8). (O) Chd is required for the anterior neural induction caused by β-Catenin. Neural and cement gland markers were induced in animal cap explants by activation of β-Catenin signal by the injection of 600 pg *β-catenin* mRNA, *dnGSK3* mRNA, or LiCl treatment (lanes 3–5). Markers of anterior brain (*Six3, Otx2*), eye (*Rx2a*), midhindbrain boundary (*En2*), hindbrain (*Krox20*), and cement gland (*XAG*) were inhibited by *Chd-*MO (lanes 6–8). Although inhibition was not detected for the posterior neural marker *HoxB9* and the pan-neural marker *NCAM*, the neuronal marker *N-tubulin* was inhibited. *α-actin* and *α-globin* are dorsal and ventral mesoderm markers, respectively, used to show the absence of mesoderm formation, and *ODC* serves as loading control.

We next investigated whether BCNE center signals are required for the anterior CNS that forms in embryos lacking mesoderm and Spemann organizer. *CerS* mRNA was injected at the 4-cell stage and the BCNE region marked with BDA at the 64-cell stage (Figure 5A; see also Figure S1). These mesoderm-less embryos developed forebrain tissue and prominent cyclopic eyes, which were derived from the lineage-labeled BCNE cells (Figure 5B and 5C). To test whether there was a requirement for Chd in these embryos, we injected *Chd-*MO at the 2-cell stage. When Chd was knocked down, BCNE cells developed into epidermis instead of CNS (Figure 5D and 5E). Brain and eye formation could be rescued by overexpression of *Chd* mRNA lacking the region targeted by *Chd-*MO (Figure 5F and 5G).

Molecular analyses confirmed that mesoderm-less embryos injected with *Chd-*MO did not express anterior neural tissue markers such as *Otx2, Rx2a, En2,* and *Krox20* (Figure 5H, compare lanes 3 and 4). However, spinal cord (*HoxB9*) or pan-neural markers (N-tubulin, neural cell adhesion molecule [NCAM]) were still expressed, indicating that only anterior neural differentiation was eliminated by *Chd-*MO and that posterior neural induction continues to take place. We also generated a Noggin antisense morpholino oligo (*Nog-*MO) reagent, which, like *Chd-*MO, inhibited brain markers (Figure 5H, lane 5). *Nog-*MO was slightly weaker than *Chd-*MO, but even a combination of both morpholinos did not eliminate posterior neural markers (Figure 5H, lane 6). These results show that the brain tissue formed in embryos lacking mesoderm and Spemann organizer derive from BCNE cells. The formation of anterior CNS in mesoderm-less embryos requires the expression of *Chd* and *Nog* in prospective neuroectoderm.

### Neural Induction by β-Catenin Requires Chd

It has recently been discovered that microinjection of *β-catenin* mRNA is able to induce neural tissue in *Xenopus* animal caps (Baker et al. 1999). Stabilization of β-Catenin has a dual effect, inhibiting the transcription of BMPs (Baker et al. 1999; Leung et al. 2003) and increasing expression of the BMP antagonists *Chd* and *Nog* in the blastula animal cap (Wessely et al. 2001). We next tested the effect of β-Catenin knockdown on CNS differentiation. As shown in Figure 5I and 5J, *β-cat-*MO oligos (Heasman et al. 2000) blocked formation of histological anterior brain and eye structures in *CerS* mesoderm-less embryos. Importantly, anterior CNS formation could be restored by overexpression of either *β-catenin* or *Chd* mRNA in these embryos (Figure 5K–5N). We conclude that brain formation in the absence of mesoderm requires the early β-Catenin signal.

To investigate whether neural induction by β-Catenin in animal cap explants required Chd, the β-Catenin pathway was activated by *β-catenin* mRNA, dominant negative glycogen synthase kinase-3 (*dnGSK3*) mRNA, or LiCl. These treatments induced multiple neural markers in animal caps (Figure 5O, lanes 3–5). Microinjection of *Chd-*MO inhibited the expression of anterior neural markers (*Six3, Otx2, Rx2a, En2*), but not of posterior or pan-neural ones (*HoxB9,* NCAM) (Figure 5O, lanes 6–8). The results indicate that neural induction by the β-Catenin signal requires expression of its downstream target gene *Chordin.*

### Anterior Neural Induction in Keller Explants Requires Chd

Is the expression of *Chd* in prospective neuroectoderm at blastula responsible for the “planar” neural induction signals (Figure 6A) described by earlier workers? To investigate this, we used Keller sandwiches (Keller and Danilchick 1988; Doniach et al. 1992; Ruiz i Altaba 1992), in which neural tissue develops without contacting underlying mesoderm (Figure 6B). Marking of the BCNE region with lineage tracer indicated that Keller sandwiches contain cells that expressed *Chd* in prospective neuroectoderm at blastula (Figure 6C). Keller sandwiches expressed anterior CNS gene markers (Figure 6D and 6F). However, explants prepared from embryos injected with *Chd-*MO failed to express *Otx2* or *Krox20* in anterior neuroectoderm, while retaining *Otx2* expression in endoderm (compare Figure 6D and 6E). N-tubulin expression, which marks differentiated neurons, was inhibited by *Chd-*MO in the anterior CNS, but persisted in prospective spinal cord (Figure 6F and 6G; results summarized in Figure 6H and 6I). These results show that the anterior CNS formation observed in Keller explants lacking underlying mesoderm requires Chd.

Molecular analyses of Keller explants confirmed that brain markers were inhibited by *Chd-*MO, while pan-neural and spinal cord markers were less affected (Figure 6J, compare lanes 2 and 3). As before, posterior neural induction did not require Chd. The origin of this posterior neural differentiation is due to fibroblast growth factor (FGF) signaling (Hongo et al. 1999; Pera et al. 2003), since it could be blocked in explants injected with dominant negative FGF receptor 4a (dnFGFR4a) mRNA (Figure 6J, lanes 4 and 5). Importantly, anterior CNS markers were still expressed in Keller sandwiches when mesoderm induction was blocked by *CerS* mRNA (Figure 6J, lane 6) and could be blocked by *Chd-*MO (Figure 6J, lane 7). Since mesoderm-less *CerS* Keller explants lack an endomesodermal Spemann organizer, their sole source of Chd is the BCNE center. Taken together, these experiments indicate that the anterior neural induction observed in Keller explants, known as “planar” induction, results from the activity of *Chd-*expressing cells located in the presumptive neuroectoderm at the blastula stage.

### Chordin and Cerberus Cooperate in Brain Induction

Do vertical signals from endomesoderm cooperate with the BCNE center in brain differentiation? The endomesoderm secretes growth-factor antagonists with head-patterning activity, such as Cer, Frzb-1, Crescent, Dickkopf-1, Chd, and Nog (Harland 2000; De Robertis et al. 2000). Several of these secreted antagonists are expressed in the anterior endoderm, which is homologous to the mouse anterior visceral endoderm (Beddington and Robertson 2000). We chose to study one of these antagonists, the head-inducer Cer, because it is expressed in the anterior endoderm of the Spemann organizer (Bouwmeester et al. 1996) and in the Nieuwkoop center, but not in the BCNE center (see Figure 1E).

**Figure 7:**
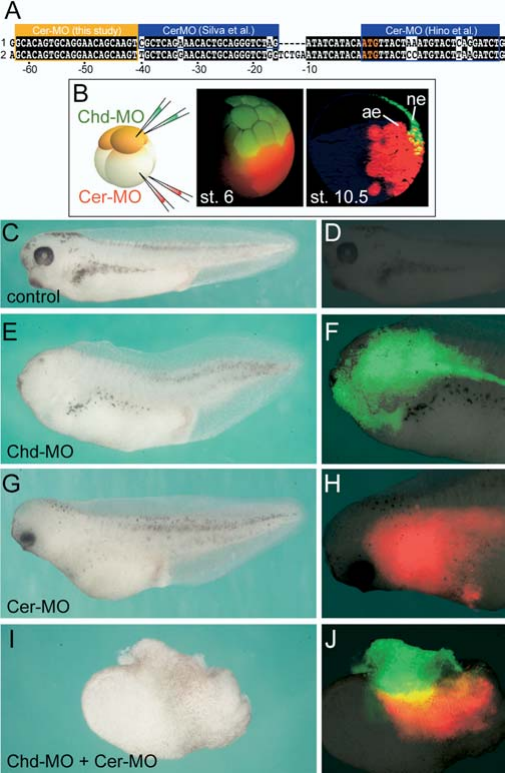
A Double-Assurance Mechanism in *Xenopus* Neural Induction That Requires Chordin and Cerberus (A) A new *Cer-*MO is complementary to both *Cer* pseudoalleles, while two MOs reported by other authors (Hino et al. 2003; Silva et al. 2003) match only one allele, having three or four mismatches, respectively, with the other allele. The *Cer-*MO used in the present study inhibits head formation in intact embryos (data not shown), while the other two do not (Hino et al. 2003; Silva et al. 2003). (B) Experimental procedure and cell lineages at 32-cell and early gastrula (stage 10.5) for dorsal-animal (FDA, green) and dorsal-vegetal (TRDA, red) blastomeres microinjected at the 8-cell stage. (C and D) Uninjected embryos. (E and F) Dorsal-animal injection with 8.5 ng of *Chd-*MO alone partially inhibited head formation; green fluorescence was seen in anterior CNS. (G and H) Dorsal-vegetal injection with 17 ng of *Cer-*MO also inhibited brain formation partially; red fluorescence may be seen in anterior endomesoderm. (I and J) Injection with 8.5 ng *Chd-*MO dorsal-animally and 17 ng *Cer-*MO dorsal-vegetally blocked brain formation, but not spinal cord and somites (histological sections not shown).

Two recent studies have described morpholino antisense oligos targeting *Cer*. In both, *Cer* did not appear to be required for head development on its own, but cooperated when coinjected with other factors (Hino et al. 2003; Silva et al. 2003). Xenopus laevis genes frequently have pseudoalleles thought to have originated from hybridization between two different *Xenopus* species in the course of evolution (Kobel and Du Pasquier 1986). Examination of the EST database showed that a second *Cer* allele existed, and that the published morpholinos had three and four mismatches with it, respectively (Figure 7A) (Silva et al. 2003; Hino et al. 2003). We therefore designed a new morpholino oligo, *Cer-*MO, targeting both X. laevis pseudoalleles (Figure 7A). *Cer-*MO inhibited head formation in *Xenopus* embryos, which could be rescued by *Cer* mRNA lacking the targeted 5′-leader sequence (data not shown).

**Figure 8:**
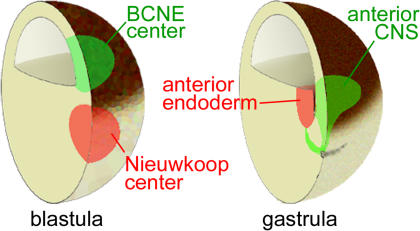
Double-Assurance Model for Brain Formation by the BCNE and Nieuwkoop Centers Blastula *Chd-* and *Nog-*expressing cells are located in the dorsal animal region, while the Nieuwkoop center is found in the dorsal-vegetal region. At gastrula, the anterior endoderm derived from the Nieuwkoop center is found in close apposition to the prospective anterior CNS. See text for discussion.

To test whether Cer and Chd cooperated, we targeted *Cer-*MO to dorsal endomesoderm and *Chd-*MO to dorsal neuroectoderm at the 8-cell stage (Figure 7B). At the doses used, microinjection of *Chd-*MO or *Cer-*MO alone resulted in partial reductions of the anterior CNS (Figure 7C–7H). However, when neuroectodermal and endomesodermal progenitor cells were injected with *Chd-*MO and *Cer-*MO, respectively, brain differentiation did not occur (Figure 7I and 7J). In histological sections, these embryos lacked brain structures but still developed spinal cord, somites, and notochord (data not shown). The results suggest that formation of the brain requires partly overlapping distinct factors in different cell layers. Chd is required in prospective neuroectoderm to predispose cells to form anterior CNS and cooperates with vertical signals from the underlying endomesoderm that include Cer (Figure 8).

## Discussion

The results presented here are consistent with the following sequence of events during CNS development in *Xenopus*. A dorsal β-Catenin signal triggered by the early cortical rotation of the egg (Gerhart et al. 1991; De Robertis et al. 2000) induces the expression of anti-BMP molecules such as Chd and Nog in a group of cells located in the dorsal animal region at the blastula stage (see Figure 8). The dorsal prospective neuroectoderm is already specified to form CNS at blastula (see Figure 3). Remarkably, transplantation studies showed that this BCNE center was required for brain formation in vivo (see Figure 4). The normal fate of these dorsal animal cap cells during development is to give rise to anterior CNS, floor plate, and notochord (see Figure 2). We note that our previous term “preorganizer” (Wessely et al. 2001) was somewhat inadequate. Since BCNE grafts are unable to induce CNS in neighboring cells after transplantation, they lack organizer activity.

The Nieuwkoop center arises at the same stage as the BCNE center, but in more vegetal cells (see Figure 8). The Nieuwkoop center expresses secreted factors such as mesoderm-inducing *Xnrs* and *Cer* and in later development gives rise to the endoderm that underlies the anterior CNS (see Figure 8). By inhibiting Chd in presumptive neuroectoderm and Cer in the endomesodermal substratum (see Figure 7), we were able to provide evidence that partly overlapping functions of distinct growth-factor antagonists secreted by different germ layers cooperate in CNS formation.

### Neural Induction Starts at Blastula

At the blastula stage, gene expression in the BCNE region causes a neural predisposition in the prospective brain tissue itself. When prospective neuroectoderm is explanted at blastula and cultured in the absence of mesoderm, it can develop into histotypic neural tissue (see Figure 3D). Once the Nieuwkoop center induces a Spemann organizer in dorsal mesoderm, a cocktail of growth factor antagonists is secreted by the endomesoderm. These Spemann organizer molecules require Nodal-related signals in order to be produced at gastrula (Agius et al. 2000; Wessely et al. 2001). These endomesodermal factors include Cer (an inhibitor of Nodal, Wnt, and BMP signals); anti-Wnts such as Frzb-1, Crescent, and Dickkopf, and anti-BMPs such as Follistatin, Chd, and Nog (De Robertis et al. 2000). Some molecules, like Chd and Nog, are expressed both in the dorsal animal cap at blastula and in the Spemann organizer at gastrula, whereas others, like Cer, are expressed only in the Nieuwkoop center and its endomesodermal descendants. In amphibians, the ability of a gastrula dorsal lip to induce a complete CNS when transplanted into a host gastrula had been taken as an indication that neural induction occurs at gastrula. We now show that brief expression of Chd and Nog during blastula stages, triggered by the maternal β-Catenin signal, is required for brain formation. This requirement becomes apparent when additional signals from underlying endomesoderm are blocked by inhibition of Nodal signaling or by preventing involution in Keller dorsal explants. We conclude that *Xenopus* neural induction starts in presumptive neuroectoderm shortly after midblastula. This is in agreement with other recent work in *Xenopus* (Baker et al. 1999; Gamse and Sive 2001; Wessely et al. 2001).

### Redundant Signals in Neural Induction

Multiple secreted growth factors antagonists participate in CNS induction (Harland 2000; De Robertis et al. 2000), and their activities can be redundant. In the mouse, *Chd* and *Nog* mutants have normal neural plates, but in *Chd* ^-/-^;*Nog* ^-/-^ embryos, development of the forebrain fails (Bachiller et al. 2000). In *Xenopus* and zebrafish, loss of Chd in the whole embryo results in animals that still are able to form anterior CNS, although its size is reduced (Schulte-Merker et al. 1997; Oelgeschläger et al. 2003). This contrasts with the strong requirement for Chd revealed here when neural induction is driven by a single signaling center in *Xenopus*. In mesoderm-less embryos, (*CerS-*injected) blastula dorsal animal cells are the sole source of Chd, and anterior CNS differentiation can be completely inhibited by *Chd-*MO (see Figure 5). Chd is also required for the neural inducing activity of Spemann organizer grafts (Oelgeschläger et al. 2003). Multiple neural-inducing molecules, such as the above-mentioned growth factor antagonists, have been identified and may compensate for the loss of Chd in intact embryos. In addition other signals located outside of dorsal signaling centers, such as FGFs and insulin-like growth factors (IGFs) may participate in neural induction (see below).

Could redundant signals from prospective neuroectoderm and endomesoderm also function in neural induction in other vertebrates? This seems possible in the case of the chick embryo. Chick *Chd* is initially expressed in the unincubated egg in epiblast central cells just anterior to Koller's sickle (Streit et al. 1998), a region that may correspond to the *Xenopus* blastula *Chd*-expressing region. The progeny of this region of the chick epiblast contributes to the prospective forebrain and moves anteriorly during development. The descendants of the chick early *Chd*-expressing brain progenitors migrate at all times in front of the organizer, which is located at the tip of the primitive streak and has a caudalizing influence (Foley et al. 2000). In zebrafish, mutant embryos lacking Nodal signaling still form brain tissue in the absence of a mesodermal organizer and express *Chd* (Gritsman et al. 1999). In the mouse embryo, transplantation experiments at the gastrula stage support a role for different germ layers in brain induction (Tam and Steiner 1999). However, expression of mouse *Chd* and *Nog* has only been analyzed from early primitive streak stage on (Bachiller et al. 2000). Studies on the expression of these BMP antagonists in prestreak or peri-implantation mouse embryos, or on the earliest nuclear localization of β-Catenin protein, will be required to determine whether a region homologous to that of the *Xenopus* blastula *Chd-*expressing region exists in mammalian embryos.

### Neural-Inducing Signals in Chordates

In amphibians the default model of neural induction proposes that BMPs expressed in ectoderm cause epidermal induction. When animal cap cells are dissociated, they become neuralized (reviewed by Weinstein and Hemmati-Brivanlou 1999). When exogenous BMP is added to dissociated animal cap cells, epidermal differentiation is restored. The present work with morpholinos that inhibit Chd, Nog, and Cer highlights the importance of BMP signaling regulation in *Xenopus.* The BCNE center appears shortly after midblastula and is required for anterior CNS formation when endomesodermal signals are inhibited. Induction of posterior neural tissue can still take in the absence of Chd and Nog (e.g., Figure 5H). Formation of this posterior neural tissue can be blocked by dominant-negative FGF receptor 4a (see Figure 6J). In *Xenopus,* FGF and IGF signaling are able to induce neural differentiation in animal caps (Hardcastle et al. 2000; Pera et al. 2001; Richard-Parpaillon et al. 2002), and late canonical Wnt signals are known to inhibit anterior brain formation (Kiecker and Niehrs 2001).

In chick and ascidian embryos, current models of neural induction highlight the role of FGF and Wnt in neural induction and de-emphasize a role for BMP regulation (Wilson and Edlund 2001; Stern 2002; Bertrand et al. 2003). We are unable to discuss in depth here the relative importance of the different signaling pathways in various organisms (reviewed in Wilson and Edlund 2001). It is clear, however, that multiple pathways cooperate in neural development. For example, in the chick embryo, Wnt or BMP antagonists applied to cells at the border region between epidermis and CNS expand the neural plate, and FGF signaling represses BMP4 expression in the neuroectoderm. In addition, the anti-neural effects of intermediate levels of an FGF antagonist can be reversed by the addition of chick *Chd* (Wilson and Edlund 2001). One of the difficulties in comparing neural induction between *Xenopus* and other chordates concerned the different timing of events. We now find a requirement for critical signals triggered by β-Catenin in the prospective neuroectoderm just after midblastula. Thus, the neural induction process seems to start at blastula in all chordates (Wessely et al. 2001; Wilson and Edlund 2001; Stern 2002; Bertrand et al. 2003). In addition, new molecular mechanisms are being discovered that help explain how disparate signaling pathways—such as those of FGF, IGF, and anti-BMPs—can be integrated during development. Tyrosine kinase receptors such as those for FGF and IGF have recently been found to inhibit the BMP pathway effector protein Smad1 by phosphorylation via mitogen-activated protein kinase (MAPK) (Pera et al. 2003; Sater et al. 2003). Neural induction by the BMP antagonist Chd requires the extra boost in Smad1 inhibition provided by FGF and IGF signaling (Pera et al. 2003). This molecular mechanism exemplifies one way in which signaling pathways hitherto considered entirely independent might be integrated in embryonic cells (Massagué 2003). Primary neural induction in the chordate embryo has been an area of active investigation for many years and we can expect this to continue for the foreseeable future.

### A Role for Ectoderm in Amphibian Neural Induction

The role of the ectoderm in amphibian neural induction has been the subject of much debate (Spemann 1938; Holtfreter and Hamburger 1955; Nieuwkoop and Koster 1995). Gene marker studies in *Xenopus* had noticed a predisposition of dorsal ectoderm for neural induction by mesoderm (Sharpe et al. 1987; London et al. 1988), but a requirement for any specific genes had not been addressed. In addition, it was known that the dorsal animal cap responds much better to the mesoderm-inducer Activin (Sokol and Melton 1991). These earlier findings can now be reinterpreted as reflecting the effects of the early β-Catenin signal that induces expression of genes such as *Chd, Nog, Xnr3,* and *Siamois. Chd* is not only expressed in the organizer region during gastrulation, but also in the dorsal animal cap region during blastula, and this is required for neural specification.

In this study we have provided evidence that presumptive neural plate material can differentiate into CNS in the absence of a mesodermal substratum. The BCNE center is required for brain formation in the embryo, but requires the cooperation of endomesodermal signals such as Cerberus. A requirement of gastrula prospective neuroectoderm for neural plate formation had been proposed by earlier workers on the basis of defect experiments (Goerttler 1925; Lehmann 1928). However, these results were disputed (Holtfreter 1933; Spemann 1938; Holtfreter and Hamburger 1955; Hamburger 1988; Nieuwkoop and Koster 1995), and vertical induction by the endomesodermal Spemann organizer was attributed the preeminent role in amphibian neural induction. A possible explanation for why the role of the prospective neuroectoderm remained unrecognized for so many years of research on the experimental embryology of neural induction is that, unlike Spemann's organizer, the BCNE center lacks inducing activity when transplanted to ectopic sites. The availability of new tools to investigate the function of individual genes—such as *β-catenin, Chd,* and *Cer*—has now provided evidence that both ectodermal and endomesodermal signals are required for primary embryonic induction in *Xenopus.*

## Materials and Methods

### 

#### Embryo manipulations

*Xenopus* embryos obtained by in vitro fertilization were cultured in 0.1× modified Barth's medium (Sive et al. 2000). For BCNE transplantation and deletion experiments, dissections were performed in 1× Steinberg's solution (Sive et al. 2000). BCNE grafts were 0.3 mm squares isolated from the dorsal animal cap just above the floor of the blastocoel. They were excised at early stage 9 (6.75–7.25 h after fertilization at room temperature), before extensive epiboly movements begin, just one division after the large-cell blastula stage (stage 8), and could be monitored by the thickness of the animal cap and cell size. Embryos were cultured in 1× Steinberg's solution until healing (0.5–1 h) and then changed into 0.1× Barth's solution. Embryo stages were according to Nieuwkoop and Faber (1994).

Keller sandwiches were prepared at early stage 10. The dorsal sector of the gastrula was excised at an angle of 30° from the dorsal midline, from the dorsal lip up to the animal pole, using stainless steel forceps. Two explants were sandwiched and cultured in 1 x Steinberg solution for 12 h for in situ hybridization, 1 d for RT-PCR analysis, and 2 d for morphological analysis. For RT-PCR analyses, RNA was pooled from five Keller explants, five animal caps, or single embryos. The RT-PCR conditions and primers, as well the protocol for whole-mount in situ hybridization, are described in http://www.hhmi.ucla.edu/derobertis/index.html.

#### Lineage tracing

To fate map BCNE descendants, an improved lineage tracing method was developed. Embryos were injected with 1–4 nl of 1% BDA (Molecular Probes, Eugene, Oregon, United States) in H_2_O and cultured explants or embryos were fixed for at least 1 h in MEMFA (Sive et al. 2000). Subsequently, embryos were placed for 24 h in 70% ethanol, 1 h in 100% ethanol, 1 h in 100% isopropanol, 12–16 h in 100% xylene, and 1 h in paraffin at 65°C before embedding. We found that overnight incubation in xylene improved sectioning of early embryos, which are rich in yolk. Sections were cut at 8–10 μm and dewaxed in 100% xylene, 100% ethanol, and 70% ethanol for 2 min each. Next, sections were washed twice in binding buffer (100 mM Tris–HCl, 150 mM NaCl [pH 7.5]) for 5 min and incubated in binding buffer containing streptavidin-coupled alkaline phospatase (Roche, Basel, Switzerland) at a dilution of 1:5,000 overnight at room temperature. Afterwards, slides were washed twice with binding buffer, once with reaction buffer (100 mM Tris–HCl, 100 mM NaCl, 50 mM MgCl_2_, pH 9.5) for 5 min and incubated overnight with reaction buffer containing 10% BM purple solution (Roche) in a Coplin jar in the dark at 4°C. Staining was stopped by incubation in Stop solution (100 mM Tris-HCl, 1 mM EDTA, pH 7.4), and sections dehydrated in 100% methanol and completely air-dried before mounting in Vectashield medium (Vector Laboratories Inc., Burlingame, California, United States). In some experiments nuclear *lacZ* mRNA (kind gift of R. Harland), fluorescein dextran amine or Texas red dextran amine were used as lineage tracers.

#### RNA injections

To generate synthetic mRNAs, the plasmids pCS2-*CerS,* pCS2-*Chd,* pCS2-*β-catenin,* and pCS2-*dnGSK3* were linearized with NotI and transcribed with SP6 RNA polymerase as described previously (Piccolo et al. 1999). The following amounts of mRNA were used for microinjections: 600 pg (150 pg four times into the vegetal region at 4-cell stage) for *CerS,* 100 pg (50 pg twice into dorsal-animal region at 4-cell stage) for *Chd,* 800 pg (400 pg twice into the dorsal-animal region at 8-cell stage) for *β-catenin,* and 600 pg (300 pg twice into the dorsal-animal region at 8-cell stage) for *dnGSK3* mRNA.

#### Morpholino oligos

Morpholino oligos were as follows: *Chd-*MO1 (5′-ACG TTC TGT CTC GTA TAG TGA GCG T-3′) and *Chd-*MO2 (5′-ACA GCA TTT TTG TGG TTG TCC CGA A-3′) (Oelgeschläger et al. 2003); *Nog-*MO (5′-TCA CAA GGC ACT GGG AAT GAT CCA T-3′) (this work); *β-cat-*MO (5′-TTT CAA CCG TTT CCA AAG AAC CAG G-3′) (Heasman et al. 2000); *Cer-*MO (5′-ACT TGC TGT TCC TGC ACT GTG C-3′) (this work); and a control-MO (5′-CCT CTT ACC TCA GTT ACA ATT TAT A-3′) (Oelgeschläger et al. 2003). The morpholino oligos were resuspended to prepare a 1 mM stock solution (SS) that was then further diluted in sterile water to give a working solution: *Chd-*MO solution (*Chd-*MO1-SS:*Chd-*MO2-SS:H_2_O = 1:1:6), *β-cat-*MO solution (*β-cat-*MO-SS:H_2_O = 1:4), *Nog-*MO solution (*Nog-*MO-SS:H_2_O = 1:1), *Cer-*MO solution (*Cer-*MO-SS:H_2_O =1:1), control-MO solution (control-MO-SS:H_2_O = 1:1), and *Chd-*MO/*Nog-*MO solution (*Chd-*MO1-SS:*Chd-*MO2-SS:*Nog-*MO-SS:H_2_O = 1:1:4:10). A total of 8 nl (two times 4 nl or four times 2 nl) morpholino solutions were injected at the 2-cell stage or 4 nl (two times 2 nl) of morpholino solution injected at the 8-cell stage.

## Supporting Information

Figure S1 The BCNE Region Can Be Reliably Marked at the 64-Cell Stage Using an Improved BDA Lineage-Tracing Method (A–H) Microinjection of individual 32-cell blastomeres does not faithfully recapitulate the lineage of BCNE grafts at gastrula (compare with Figure 2B and 2D). Note that the lineage of D1 includes part of the Nieuwkoop center and contributes to anterior endoderm at gastrula. This 32-cell map is in general agreement with previously published fate maps (Dale and Slack 1987; Bauer et al. 1994); the minor differences observed are explained by our choice of batches of regularly cleaving embryos (Klein 1987) with tightly adhering small animal blastomeres. Abbreviation: AE, anterior endoderm.
(I–L) Diagram indicating the injection of the lower daughter of B1 and the upper daughter of C1 at the 64-cell stage (I), which reliably identify BCNE descendants at stage 9 (J), stage 11 (K), and stage 36 (L). Arrowheads indicate the blastopore. Abbreviations: fp, floor plate; no, notochord.
(4.39 MB TIF).

### Accession Numbers

The GenBank (http://www.ncbi.nlm.nih.gov/Genbank/index.html) accession numbers discussed in this paper are for *β-catenin* (M77013), *Cer* (U64831), *Cer* pseudoallele (BG160114), *Chd* (L35764), *Goosecoid* (M81481), and *Nog* (M98807).

## Abbreviations

*β-cat-*MO: *β-catenin* morpholino oligos
BCNE: blastula *Chordin-* and *Noggin-*expressing region
BMP: bone morphogenetic protein
*Cer-*MO: *Cerberus* morpholino oligos
*CerS*: *Cerberus-Short*
*Chd-*MO: *Chordin* morpholino oligos
CNS: central nervous system
FGF: fibroblast growth factor
GSK3: glycogen synthase kinase-3
IGF: insulin-like growth factor
LiCl: lithium chloride
MO: antisense morpholino oligo
MAPK: mitogen-activated protein kinase
NCAM: neural cell adhesion molecule
*Nog*-MO: *Noggin* morpholino oligos
ODC: ornithine decarboxylase
SS: stock solution
*Xnr*: *Xenopus* Nodal-related
