# Supplementary figures and images for: Neural Induction in Xenopus: Requirement for Ectodermal and Endomesodermal Signals via Chordin, Noggin, β-Catenin, and Cerberus
Source: PLoS Biol. 2004 May 11;2(5):e92. doi: 10.1371/journal.pbio.0020092 (PMC406387; doi:10.1371/journal.pbio.0020092)

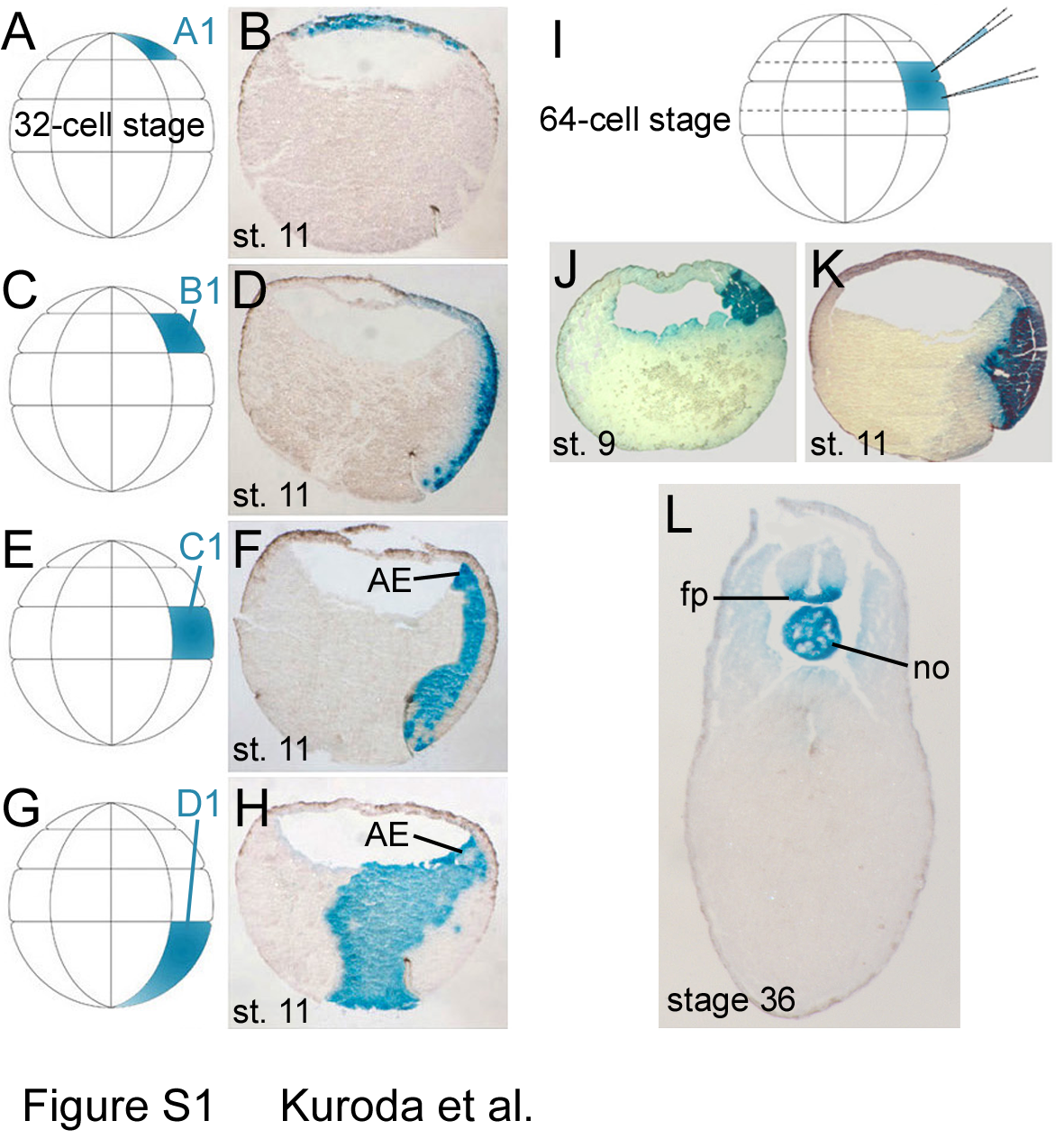

Supplement: Figure S1 — (A–H) Microinjection of individual 32-cell blastomeres does not faithfully recapitulate the lineage of BCNE grafts at gastrula (compare with Figure 2B and 2D). Note that the lineage of D1 includes part of the Nieuwkoop center and contributes to anterior endoderm at gastrula. This 32-cell map is in general agreement with previously published fate maps (Dale and Slack 1987; Bauer et al. 1994); the minor differences observed are explained by our choice of batches of regularly cleaving embryos (Klein 1987) with tightly adhering small animal blastomeres. Abbreviation: AE, anterior endoderm. (I–L) Diagram indicating the injection of the lower daughter of B1 and the upper daughter of C1 at the 64-cell stage (I), which reliably identify BCNE descendants at stage 9 (J), stage 11 (K), and stage 36 (L). Arrowheads indicate the blastopore. Abbreviations: fp, floor plate; no, notochord. (4.39 MB TIF). [file pbio.0020092.sg001.tif]
